# Preparation of a universally usable, animal product free, defined medium for 2D and 3D culturing of normal and cancer cells^☆^

**DOI:** 10.1016/j.mex.2024.102592

**Published:** 2024-02-03

**Authors:** Tilo Weber, Jeffrey Bajramovic, Stina Oredsson

**Affiliations:** a Animal Welfare Academy of the German Animal Welfare Federation, Neubiberg 85579, Federal Republic of Germany; b 3Rs Centre Utrecht, Utrecht University, Utrecht 3584 CJ, The Netherlands; c Department of Biology, Lund University, Lund 22362, Sweden

**Keywords:** Defined medium, Cell culture, Reproducible composition, Human proteins, Ethically acceptable, Animal free medium, Protocols for mixing animal product free defined cell culture medium

## Abstract

Since 1958, cell culture media supplemented with fetal bovine serum is used, despite the well-known concerns about animal welfare, reproducibility, reliability, relevance, and safety. To obliterate these concerns and increase scientific accuracy, we recently published an open access, publicly available paper on a defined medium composition to make it possible for any lab to prepare this medium. The medium supports routine culturing and cell banking as well as investigations of growth curves, dose response testing of compounds of cells in 2D and 3D, and cell migration; all important aspects for research and toxicology. Here we give a detailed description of how to mix the defined universal cell culture medium in 14 simple steps to support any entity that wishes to make it. We also list different normal and cancer cell lines that have been cultured in the defined medium.

• Open source composition of animal product free universal cell culture medium
• Protocols for mixing solutions of small xeno free molecules for supplementation
• Protocols for mixing solutions of human proteins for supplementation

## Graphical abstract

**Figure:**
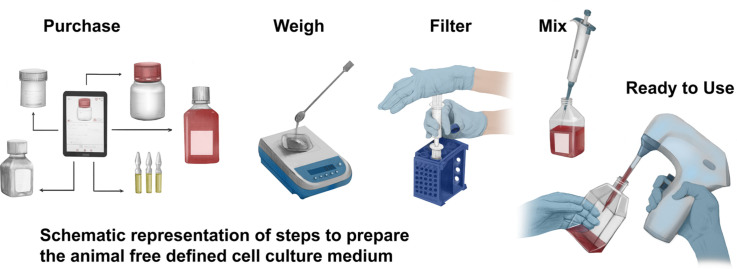

Specifications table

**Table:** 

Subject area:	Pharmacology, Toxicology and Pharmaceutical Science
More specific subject area:	Animal product free defined cell culture medium
Name of your method:	Protocols for mixing animal product free defined cell culture medium
Name and reference of original method:	A new animal product free defined medium for 2D and 3D culturing of normal and cancer cells to study cell proliferation and migration as well as dose response to chemical treatment https://doi.org/10.1016/j.toxrep.2023.04.001
Resource availability:	Vendors and product information in the protocols

## Method details

Since cells are cultured *in vitro*, the search for an optimal culture medium is ongoing [1]. So far, the use of media supplemented with mammal-based serum ingredients is an often taken choice [2], [3], [4]. These are mostly of bovine or human origins, like fetal bovine serum (FBS) and human platelet lysate (hPL), respectively. However, these supplements are undefined, characterized by batch-to-batch variability, may induce a non-physiological, proliferating cellular phenotype, and bare the risk of contamination or even infections [5,6]. Together, this negatively affects safety, relevance, and reproducibility of research outcomes [7], [8], [9]. Especially, when culturing e.g. human cells in a non-human medium like FBS-supplemented medium, the transferability of the experimental results is questionable. Furthermore, it is an ethical necessity to replace animal-derived ingredients like FBS in the laboratory to maximize animal welfare [10], [11], [12], [13].

In 2023, Rafnsdóttir *et al*. published a paper on a defined, safe to use, animal free, and universally usable cell culture medium [14]. Table 1 lists the cell lines that were used in the published paper. Table 2 shows a list of different cancer and normal cell lines that since then have been adapted to the medium and that have been routine-cultured to ascertain stable growth. Presently we are performing experiments with HeLa, MCF-7, and MCF-10A cells (not published yet). Also, a number of dose response experiments have been performed with the pancreatic cancer cells lines (not published yet).

**Table 1:** Compilation of cell lines cultured in the defined medium described in Rafnsdóttir *et al.*[14].

Cell line name	Characterisation	Provider	Product number
CaCo-2	Human colon cancer	ATCC^a^	HTB-37
Cancer-associated fibroblasts	Human fibroblasts	Kojima *et al*., 2010 [15]	—
JIMT-1	Human breast cancer	DSMZ^b^	ACC589
KeratinoSens	Human keratinocytes	acCCELLerate^c^	RE242
L929	Mouse fibroblasts	ATCC	CCL-1
MDA-MB-231	Human breast cancer	ATCC	HTB-26
MiaPaCa-2	Human pancreatic cancer	ATCC	CRL-1420

a American Type Culture Collection, Manassas, Virginia, USA. https://www.atcc.org/.

b Deutsche Sammlung von Mikroorganismen und Zellkulturen (German Collection of Microorganisms and Cell Cultures), Braunschweig, Germany. https://www.dsmz.de/.

c Hamburg, Germany. https://www.accellerate.me/.

**Table 2:** Compilation of cell lines cultured in the defined medium, unpublished.

Cell line name	Characterisation	Provider	Product number
AsPC-1	Human pancreatic cancer	ATCC	CRL-1682
BxPC-3	Human pancreatic cancer	ATCC	CRL-1687
C6	Rat glioma	ATCC	CCL-107
CaOv-3	Human ovarian cancer	ATCC	HTB-75
HDF, adult	Human dermal fibroblasts, adult	Sigma-Aldrich^a^	106-05A
HeLa	Human cervical cancer	ATCC	CRM-CCL-2
LAN-1	Human neuroblastoma	DSMZ	ACC 655
MCF-7	Human breast cancer	ATCC	HTB-22
MCF-10A	“Normal-like” human breast epithelial	ATCC	CRL-10317
NmuMg	Mouse mammary gland epithelial	ATCC	CRL-1636
PanC-1	Human pancreatic cancer	ATCC	CRL-1469

a Sigma-Aldrich Sweden AB, Stockholm, Sweden. https://www.sigmaaldrich.com/.

Here we provide a detailed protocol on how to prepare the defined medium. After the stock solutions of the components are prepared, the medium can be mixed by following only 14 simple steps. This protocol is using human-derived proteins, as well as human recombinant proteins in accordance with our publication Rafnsdóttir *et al.* [14]. However, it should be possible to use only recombinant proteins (e.g. recombinant human serum albumin [16]) to even further increase reproducibility.

In addition, although the medium has been used for a variety of cell lines and we define it as universal, we hope this can be a starting point for abandoning the use of FBS and for further refinement to more exactly reproduce the human cellular environment.

## Important information before preparation of the medium

**Fig. 1:**
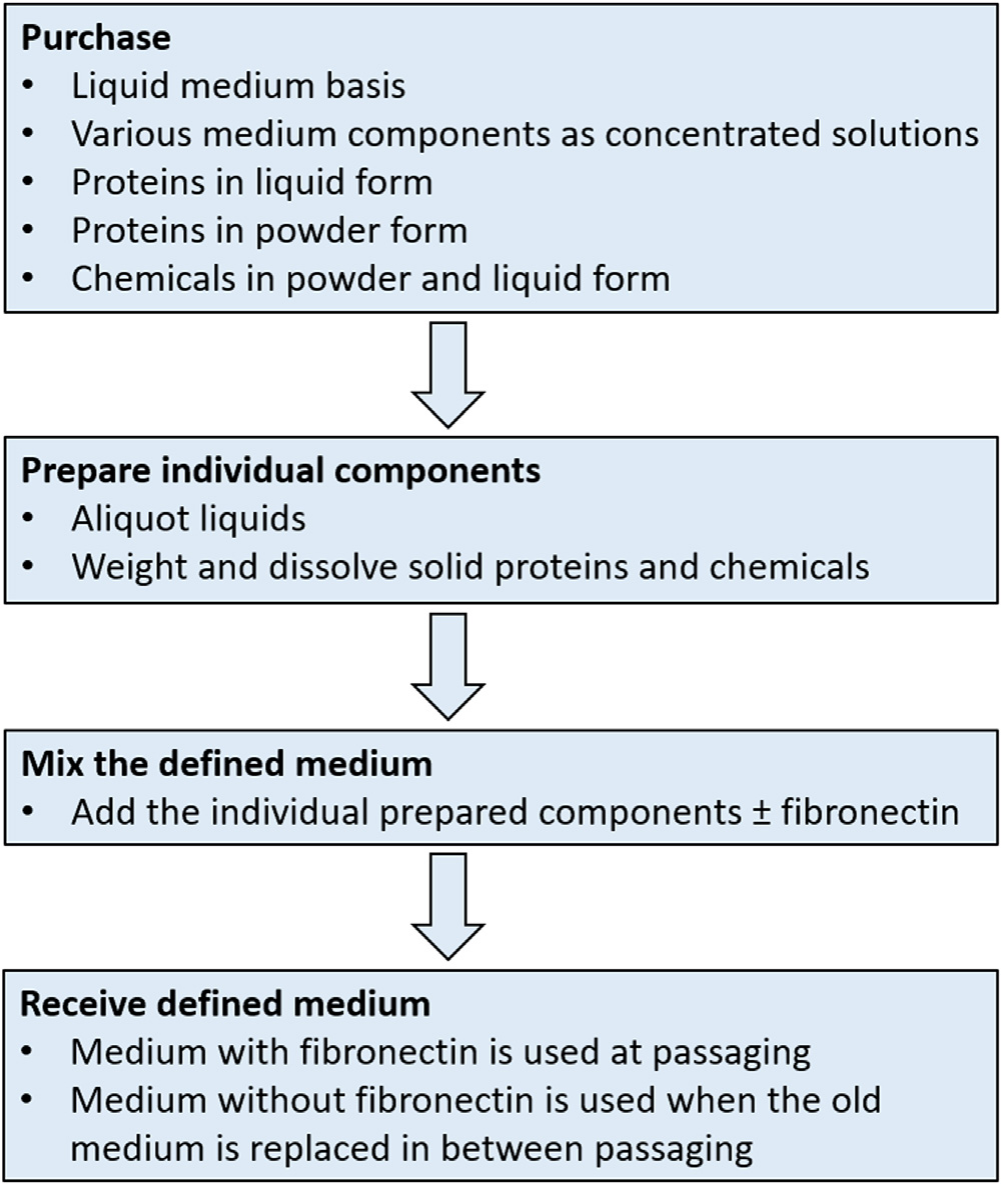
Schematic presentation of the phases of the medium preparation.

Prior to the actual mixing of the defined medium, different phases have to be taken according to Fig. 1. First, purchase all components needed for the medium. The company products displayed below are suggestions. Products with similar properties from different providers than listed here can be used for the preparation of the medium as well, e.g. human serum albumin from SeraCare^1^ (product number 1850-0028) or human placenta laminin [17] from THT Biomaterials^2^ (product number THT0201), instead of those listed below in Tables 4 and 5.

The basis for our medium is DMEM/F12, which was developed for cells in monolayer culture and has a bicarbonate content for the use in an incubator with 5% CO_2_ to obtain correct pH. Please observe that the original DMEM has a bicarbonate content that provides the correct pH when used in an incubator with 10% CO_2_. Thus, it should never be used in an incubator with 5% CO_2_, which unfortunately is found in publications and cell bank recommendations regarding medium for cell lines.

Additionally, the DMEM/F12 product mentioned here contains phenol red. Since phenol red is a xenobiotic with low estrogenic activity, a medium without phenol red would be preferred. Phenol red is added for visual pH control but with knowledge of how bicarbonate functions in pH control, phenol red can be omitted [14].

Furthermore, this medium is made for cells that require attachment and has only been tested for such cells. We have not done any studies on cells in suspension culture with the exception of human CD4^+^ T-cells that were activated by CD3/CD28 microbeads to stimulate cell proliferation in the defined medium using RPMI1640 instead of DMEM/F12 (not published). When using DMEM/F12, the final concentration of each (non-protein) component in our defined medium can be seen in Table 26. Our medium mimicks the complexity of human serum and industrial manufacturing will lower its production price significantly [24].

**Table 26:** Composition of all non-protein components in the defined medium after supplementation of DMEM/F12 to enrich the medium.

Component	Concentration found in DMEM/F12	Added supplement	Final concentration in our medium
**Amino Acids**			
Glycine	18.75 µg/ml	7.5 ng/ml	18.7575 µg/ml
L-Alanine	4.45 µg/ml	8.9 ng/ml	4.4589 µg/ml
L-Arginine hydrochloride	147.5 µg/ml	—	147.5 µg/ml
L-Asparagine monohydrate	7.5 µg/ml	15 ng/ml	7.515 µg/ml
L-Aspartic acid	6.65 µg/ml	13.3 ng/ml	6.6633 µg/ml
L-Cysteine hydrochloride monohydrate	17.56 µg/ml	—	17.56 µg/ml
L-Cystine dihydrochloride	31.29 µg/ml	—	31.29 µg/ml
L-Glutamic acid	7.35 µg/ml	14.7 ng/ml	7.3647 µg/ml
L-Glutamine	—	2 mM	2 mM
L-Histidine hydrochloride monohydrate	31.48 µg/ml	—	31.48 µg/ml
L-Isoleucine	54.47 µg/ml	—	54.47 µg/ml
L-Leucine	59.05 µg/ml	—	59.05 µg/ml
L-Lysine hydrochloride	91.25 µg/ml	—	91.25 µg/ml
L-Methionine	17.24 µg/ml	—	17.24 µg/ml
L-Phenylalanine	35.48 µg/ml	—	35.48 µg/ml
L-Proline	17.25 µg/ml	11.5 ng/ml	17.2615 µg/ml
L-Serine	26.25 µg/ml	10.5 ng/ml	26.2605 µg/ml
L-Threonine	53.45 µg/ml	—	53.45 µg/ml
L-Tryptophan	9.02 µg/ml	—	9.02 µg/ml
L-Tyrosine disodium salt dihydrate	55.79 µg/ml	—	55.79 µg/ml
L-Valine	25.85 µg/ml	—	25.85 µg/ml
**Vitamins**			
Biotin	3.5 ng/ml	—	3.5 ng/ml
Choline chloride	8.98 µg/ml	3.5 µg/ml	12.48 µg/ml
D-Calcium pantothenate	2.24 µg/ml	—	2.24 µg/ml
Folic acid	2.65 µg/ml	0.33 µg/ml	2.98 µg/ml
*I*-Inositol	12.6 µg/ml	4.5 µg/ml	17.1 µg/ml
Niacinamide	2.02 µg/ml	—	2.02 µg/ml
Pyridoxine hydrochloride	2 µg/ml	—	2 µg/ml
Riboflavin	219 ng/ml	—	219 ng/ml
Thiamine hydrochloride	2.17 µg/ml	0.08 µg/ml	2.25 µg/ml
α-Tocopherol phosphate	—	3 ng/ml	3 ng/ml
Vitamin B12	0.68 µg/ml	0.35 µg/ml	1.03 µg/ml
**Inorganic Salts**			
Calcium chloride (CaCl_2_) anhydrous	116.6 µg/ml	—	116.6 µg/ml
Cupric sulfate pentahydrate (CuSO_4_ · 5H_2_O)	1.3 ng/ml	—	1.3 ng/ml
Ferric nitrate nonahydrate (Fe(NO_3_)_3_ · 9H_2_O)	50 ng/ml	—	50 ng/ml
Ferrous sulfate heptahydrate (FeSO_4_ · 7H_2_O)	417 ng/ml	—	417 ng/ml
Magnesium chloride (MgCl_2_) anhydrous	28.64 µg/ml	—	28.64 µg/ml
Magnesium sulfate (MgSO_4_) anhydrous	48.84 µg/ml	—	48.84 µg/ml
Potassium chloride (KCl)	311.8 µg/ml	—	311.8 µg/ml
Sodium bicarbonate (NaHCO_3_)	2438 µg/ml	—	2438 µg/ml
Sodium chloride (NaCl)	6995.5 µg/ml	—	6995.5 µg/ml
Sodium phosphate dibasic (Na_2_HPO_4_) anhydrous	71.02 µg/ml	—	71.02 µg/ml
Sodium phosphate monobasic monohydrate (NaH_2_PO_4_ · H_2_O)	62.5 µg/ml	—	62.5 µg/ml
Zinc sulfate heptahydrate (ZnSO_4_ · 7H_2_O)	432 ng/ml	—	432 ng/ml
**Other Components**			
4-Aminobenzoic acid	—	12 ng/ml	12 ng/ml
All-*trans*-retinoic acid	—	25 ng/ml	25 ng/ml
Ascorbic acid	—	12 ng/ml	12 ng/ml
Cholesterol	—	50 ng/ml	50 ng/ml
Dextrose (D-Glucose)	3151 µg/ml	—	3151 µg/ml
β-Estradiol	—	0.5 pg/ml	0.5 pg/ml
Glutathione	—	12 ng/ml	12 ng/ml
Hydrocortisone	—	0.25 ng/ml	0.25 ng/ml
Hypoxanthine monosodium	2.39 µg/ml	—	2.39 µg/ml
Lipoic acid	105 ng/ml	50 ng/ml	155 ng/ml
Linoleic acid	—	1 µg/ml	1 µg/ml
*O*-Phosphorylethanolamine	—	5 µg/ml	5 µg/ml
Phenol red	8.1 µg/ml	—	8.1 µg/ml
Putrescine dihydrochloride	81 ng/ml	—	81 ng/ml
Ribose	—	125 ng/ml	125 ng/ml
Selenous acid	—	8 ng/ml	8 ng/ml
Sodium pyruvate	0.5 mM	1 mM	1.5 mM
Thymidine	365 ng/ml	—	365 ng/ml
Triiodothyronine	—	0.2 pg/ml	0.2 pg/ml
Uracil	—	75 ng/ml	75 ng/ml
Xanthine	—	85 ng/ml	85 ng/ml

Instead of coating the culture surfaces with fibronectin separately, we add fibronectin directly to the culture medium. Hence, when cells are seeded in the defined medium with fibronectin at passaging, the tissue culture surface will be covered with sufficient amounts of fibronectin. This also means that for the replacement medium in between passaging, use the medium without fibronectin as described in Rafnsdóttir *et al.* [14]. We have found that many cell lines do not appear to thrive when fibronectin is present in replacement medium.

Most cell culture laboratories already purchase concentrated solutions of cell culture components such as glutamine, sodium pyruvate, non-essential amino acid, and penicillin-streptomycin. We always aliquot these in 5 ml portions, which results in the desired concentration in 500 ml medium and keep the tubes frozen at −20 °C. If possible, it is good to avoid antibiotics like penicillin-streptomycin as they also are xenobiotics. The half-life of antibiotics is quite short at 37 °C, about 2 days for penicillin and about 4 days for streptomycin. When sampling medium for mycoplasma testing, the cells should be cultured for 2 weeks in antibiotics free medium. This is a test of good sterile technique and possibly stimulates the constant use of medium without antibiotics.

Some proteins needed for the defined medium are purchased as solutions and we suggest aliquoting as seen below.

When all solutions are prepared, thawed, and placed in the laminar air-flow (LAF) bench, the final mixing takes approximately 30 min for a person that is used to pipetting. Do not turn on the light in the LAF bench. As can be found in the protocols below, some components are light sensitive, and thus, the entire medium is light sensitive. The light sensitivity of media has been tested and reported and in general, all media are light sensitive [18,19]. Vitamins and retinoic acid mainly contribute to the light sensitivity [20], [21], [22]. We have not performed a systematic investigation of the light sensitivity of the defined medium but rely on published data. We have never had problems working in the LAF bench with the light off and have used the medium in courses with undergraduate students with no problems or complaints.

The complete medium is stored at 4 °C and we recommend that it is used within two months. Freezing the medium at −20 °C will extend this approximately to four months; however, we have not performed a systematic storage time test of the complete defined medium a −20 °C. Below you will find more information about aliquoting the mixed medium to minimize pH changes as well as the possibility of degradation of components with repeated warming of the medium before addition to cells.

## Recommended tissue culture plastic

Most tissue culture plastic is made of polystyrene, although this material is naturally hydrophobic. It therefore poorly supports cell adhesion and cells that require attachment will die. Tissue culture plastic for cell culturing is oxygen plasma-treated resulting in the insertion of oxygen containing groups in the styrene molecules. In contact with water, ionization results in a negatively charged surface [23].

Our experience shows that cells thrive better in the defined medium when cultured on Corning^3^ Primaria tissue culture plastic [14]. The reason is that these plastics contain ammonium groups besides the oxygen containing groups and therefore become both positively and negatively charged in contact with water.

## Mixing of the defined medium with prepared stock solutions

This is a description of how to combine the different components (see Table 3, Table 4, Table 5) with the respective solvents (see Table 6). Enclosed are individual protocols for different components. The final mixing takes approximately 30 min for a person that is used to pipetting when all components are thawed and placed in the LAF bench. Do not turn on the light in the LAF bench. The medium is light sensitive.

**Table 3:** Components stored at +4 °C.

Component	Preparation	Provider	Product number
DMEM/F12 500 ml	Purchased	Biowest^a^	L0090-500
Insulin	Purchased	Sigma-Aldrich	I9278

a Nuaillé, France. https://biowest.net/.

**Table 4:** Components stored at −20 °C.

Component	Preparation	Provider	Product number
L-Glutamine	Purchased	Sigma-Aldrich	G7513-100ML
Non-essential amino acids (NEAA)	Purchased	Sigma-Aldrich	M7145-100ML
Sodium pyruvate	Purchased	Sigma-Aldrich	S8636-100ML
Antibiotics (penicillin-streptomycin)	Purchased	Sigma-Aldrich	P0781-100ML
Fibronectin	See Table 18: Preparation of the fibronectin solution.	EMD Millipore Corporation^a^	FC010-10MG
Transferrin	See Table 23: Preparation of the transferrin solution.	Sigma-Aldrich	T3705-1G
Human serum albumin (HSA)	See Table 25: Preparation of the human serum albumin (HSA) solution for the defined medium and Table 19: Preparation of PBS with 0.1 % human serum albumin (HSA).	Biowest	P6140

a Subsidiary of Merck KGaA, Darmstadt, Germany. https://www.emdmillipore.com/.

**Table 5:** Components stored (aliquoted in Eppendorf tubes together in plastic bags) at −80 °C.

Component		Preparation	Provider	Product number
Ethanol solution	4-Aminobenzoic acid	See Table 7: Preparation of the ethanol solution.	Sigma-Aldrich	A9878
	Cholesterol		Sigma-Aldrich	C3045
	Lipoic acid		Sigma-Aldrich	07039-10MG
	Linoleic acid		Sigma-Aldrich	L1012
NaOH solution	Folic acid	See Table 8: Preparation of the NaOH solution.	Sigma-Aldrich	F8758
	Uracil		Sigma-Aldrich	U1128
	Xanthine		Sigma-Aldrich	X3627
H_2_O solution	Ascorbic acid	See Table 9: Preparation of the H_2_O solution.	Sigma-Aldrich	A4403
	Choline chloride		Sigma-Aldrich	C7527
	Glutathione		Sigma-Aldrich	G6013
	*I*-Inositol		Sigma-Aldrich	I7508
	*O*-Phosphorylethanolamine		Sigma-Aldrich	P0503
	Ribose		Sigma-Aldrich	R9629
	Selenous acid		Sigma-Aldrich	211176
	Thiamine hydrochloride		Sigma-Aldrich	T1270
	α-Tocopherol phosphate		Sigma-Aldrich	T2020
	Vitamin B12		Sigma-Aldrich	V6629
All-*trans* retinoic acid		See Table 10: Preparation of the all-*trans* retinoic acid solution.	Sigma-Aldrich	R2625-50mg
β-Estradiol		See Table 11: Preparation of the β-estradiol solution.	Sigma-Aldrich	E2758-250MG
Hydrocortisone		See Table 12: Preparation of the hydrocortisone solution.	Sigma-Aldrich	H0888
Triiodothyronine		See Table 13: Preparation of the triiodothyronine solution.	Sigma-Aldrich	T6397
Basic fibroblast growth factor (bFGF)		See Table 14: Preparation of the basic fibroblast growth factor.	Sigma-Aldrich	F3685-25UG
Collagen Type 4		See Table 15: Preparation of the collagen solution.	Sigma-Aldrich	C5533-5MG
Epidermal growth factor (EGF)		See Table 16: Preparation of the epidermal growth factor solution.	Sigma-Aldrich	E9644-.2MG
Fetuin A		See Table 17: Preparation of the fetuin A (α2-hs-glycoprotein) solution.	Sigma-Aldrich	G0516-1MG
Insulin-like growth factor 1 (IGF-1)		See Table 20: Preparation of the insulin-like growth factor 1 solution.	ThermoFisher Scientific^a^	PHG0071
Platelet-derived growth factor AA (PDGF)		See Table 21: Preparation of the platelet-derived growth factor AA solution.	PeproTech^b^	100-13A
Laminin		See Table 22: Preparation of the laminin solution.	Sigma-Aldrich	L6274-.5MG
Vitronectin		See Table 24: Preparation of the vitronectin solution.	Stemcell Technologies^c^	07180

a Waltham, Massachusetts, USA. https://www.fishersci.com/.

b Cranbury, New Jersey, USA. https://www.peprotech.com/.

c Vancouver, British Columbia, Canada. https://www.stemcell.com/.

**Table 6:** Solvents needed for the preparation of the medium components. Stored at +20 °C.

Solvent	Preparation	Provider	Product number
Dimethyl sulfoxide (DMSO)	Purchased	PanReac ApliChem ITW Reagents^a^	A3672,0100
Ethanol gradient grade	Purchased	Sigma-Aldrich	1.11727
Phosphate-buffered saline (PBS)	Prepare and sterile filter PBS		
Sodium hydroxide (NaOH)	Prepare NaOH freshly		
Sterile Millipore H_2_O	Sterilize it by autoclaving or sterile filtering		

a Monza, Italy. https://itwreagents.com/.

*Medium components needed in the Laminar Air-Flow (LAF) bench*

Please note that ergocalciferol, as mentioned in the original medium description [14] was found to be dispensable and can be dismissed.

### Materials needed in the LAF bench

Automatic pipettes and tips to pipet different volumes from 10 µl to 250 µl.

Pipettes 5 and 10 ml (preferably glass pipettes to reduce environmental impact).

Sterile bottles for medium aliquoting and for collecting surplus DMEM/F12.

### Procedure

1. Remove 43.7 ml of the 500 ml DMEM/F12 (to compensate for added volumes). Save this surplus separately in a sterile flask. Mark well! You can collect medium in the same flask until you have enough to make more defined medium.
2. Add 5 ml 200 mM L-glutamine.
3. Add 5 ml 10 mM NEAA giving.
4. Add 5 ml 100 mM sodium pyruvate.
5. Add 5 ml penicillin-streptomycin (optional). If this is not used, re-add 5 ml of DMEM/F12.
6. Add 2.5 ml transferrin solution.
7. Add 100 µl insulin solution.
8. Turn off the light in the LAF bench.
9. Thaw the Eppendorf tubes kept at −80 °C in the dark until they reach room temperature. Spin at 2000 *g* for 30 s before pipetting to the medium.
10. Add 20 ml of the HSA solution. Mix well.
11. Now we usually divide the medium into two sterile 250 ml bottles. Label one bottle: “Defined medium without fibronectin for medium replacement” plus date. Caution: Fibronectin needs to be thawed slowly without disturbance at 37 °C to prevent clumping/precipitation.
12. Label the other bottle: “Defined medium with fibronectin for passaging” plus date. Add 250 µl fibronectin solution to this flask. Keep the remaining fibronectin at 4 °C. It can be stored at 4 °C for 3 months.
13. If you decide to make 500 ml defined medium with fibronectin, skip the steps 11 and 12 and add 500 µl of the fibronectin solution instead.
14. Store at 4 °C for a maximum of 2 months or freeze at −20 °C for 4 months.

## Mixing of solutions with small organic and inorganic molecules

A number of proteins and chemicals are purchased as powders and below are protocols for making solutions. An important part of making solutions from powdery compounds is the possibility to weight with a high precision scale to reach a satisfactory accuracy and reproducibility. The scale we use has a precision of 0.00001 g. **However, we recommend not weighing less than 100** **µg**.

Please note that before weighing laboratory materials stored below room temperature, allow these to come to room temperature. Otherwise, mass errors could occur due to condensation.

Be beware of the fact, that weighing small and precise masses of solids is more challenging than pipetting small and precise volumes of liquids. Therefore, to keep the concentration at the desired level, it might be easier to add the liquid amount according to the weighed solid mass. It is preferable to weigh slightly more than the exact amounts suggested below in the protocols, to not fall below the needed volume for the subsequent steps.

For instance: To receive a desired concentration of 0.2 µg/µl, you need to add precisely 1000 µg of solid Y to precisely 5000 µl of liquid X. If you have weighed 1094 µg of solid Y instead, you could receive the exact same desired concentration by adding 5470 µl of liquid X.

Example of calculating the volume:

$$\frac{1000\;\mu g}{5000\;\mu l}\;=\;\frac{1094\;\mu g}{X\;\mu l}$$

$$X\;\mu l\;=\;\frac{1094\;\mu g\;*\;5000\;\mu l}{1000\;\mu g}$$

X=5470μl

Keep this in mind when preparing all compounds that need weighing.

## How to prepare the stock solutions

Here you can find the protocols for the separate stock solutions. Once they are prepared, you just have to thaw and mix them according to the 14-step procedure mentioned above. An excel sheet for ticking off compounds while mixing the medium can be found in the supplementary materials.

**Table 7:** Preparation of the ethanol solution.

Product	Supplemented concentration	Amount in the aliquot (25 µl)
4-Aminobenzoic acid	12 ng/ml	6.0 µg
Cholesterol	50 ng/ml	25 µg
Lipoic acid	50 ng/ml	25 µg
Linoleic acid	1 µg/ml	500 µg
**General Information**		
These compounds are soluble in gradient grade ethanol. Thus, start by preparing a stock solution for each compound for receiving the supplemented concentration in 500 ml defined medium as shown above.		
**Preparation** 1. 4-Aminobenzoic acid: Weigh 600 µg 4-aminobenzoic acid and dissolve in 200 µl 99.9 % ethanol, yielding a desired concentration of 3 µg/µl. You will use **200** **µl** in step 5. 2. Cholesterol: Weigh 2500 µg of cholesterol and dissolve in 500 µl 99.9 % ethanol, yielding a desired concentration of 5 µg/µl. You will use **500** **µl** in step 5. 3. Lipoic acid: Weigh 2500 µg of lipoic acid and dissolve in 500 µl 99.9 % ethanol, yielding a desired concentration of 5 µg/µl. You will use **500** **µl** in step 5. 4. Linoleic acid: This is an oil with a density of 902 µg/µl at 25 °C. Take 55.4 µl of the oil to a sterile test tube. 5. Add the volumes in **bold** to the tube with linoleic acid. This will result in 1255.4 µl solution. Add 1244.6 µl 99.9 % ethanol, resulting in a total volume of 2500 µl. 6. Sterile filter. 7. Label the side of sterile Eppendorf tubes with “Ethanol Solution”. Mark the lid with ES. The solution made is for 100 tubes but we suggest preparing 50 tubes. 8. Aliquot in the sterile Eppendorf tubes (25 µl per tube). 9. Store at −80 °C.

**Table 8:** Preparation of the NaOH solution.

Product	Supplemented concentration	Amount in the aliquot (50 µl)
Folic acid	330 ng/ml	165 µg
Uracil	75 ng/ml	37.5 µg
Xanthine	85 ng/ml	42.5 µg
**General Information**		
These compounds are soluble in NaOH. Thus, start by preparing a stock solution for each compound for receiving the supplemented concentration in 500 ml defined medium as shown above. Then combine these according the instructions.		
**Preparation** 1. Folic acid: Weigh 16.5 mg folic acid and dissolve in 500 µl 0.5 M NaOH, yielding a desired concentration of 33 µg/µl. You will use **500** **µl** in step 5. 2. Uracil: Weigh 3.75 mg uracil and dissolve in 500 µl 0.5 M NaOH, yielding a desired concentration of 7.5 µg/µl. You will use **500** **µl** in step 5. 3. Xanthine: Weigh 4.25 mg xanthine and dissolve in 1000 µl 1 M NaOH, yielding a desired concentration of 4.25 µg/µl. You will use **1000** **µl** in step 5. 4. Add the volumes in **bold** to a new test tube. This will result in 2 ml solution. Add 3 ml of 0.5 M NaOH, resulting in a total volume of 5000 µl. 5. Sterile filter the solution. 6. Label sterile Eppendorf tubes with “NaOH solution”. Mark the lid with a N. The solution made is for 100 tubes but we suggest preparing 50 tubes. 7. Aliquot in the sterile Eppendorf tubes (50 µl per tube). 8. Store at −80 °C.

**Table 9:** Preparation of the H_2_O solution.

Product	Supplemented concentration	Amount in the aliquot (200 µl)
Ascorbic acid	12 ng/ml	6 µg
Choline chloride	3.5 µg/ml	1.75 mg
Glutathione	12 ng/ml	6 µg
*I*-Inositol	4.5 µg/ml	2.25 mg
*O*-Phosphorylethanolamine	5 µg/ml	2.5 mg
Ribose	125 ng/ml	62.5 µg
Selenous acid	8 ng/ml	4 µg
Thiamine hydrochloride	80 ng/ml	40 µg
α-Tocopherol phosphate	3 ng/ml	1.5 µg
Vitamin B12	0.35 µg/ml	175 µg
**General Information**		
These compounds are soluble in H_2_O. Thus, start by preparing a stock solution for each compound for receiving the supplemented concentration in 500 ml defined medium as shown above. Then combine these according the instructions. Some compounds are light sensitive. Work under subdued light.		
**Preparation** 1. Ascorbic acid: Weigh 600 µg folic acid and dissolve in 500 µl sterile Millipore H_2_O, yielding a desired concentration of 1.2 µg/µl. You will use **500** **µl** in step 11. 2. Choline chloride: Weigh 175 mg choline chloride and dissolve in 3000 µl sterile Millipore H_2_O, yielding a desired concentration of 58.3 µg/µl. You will use **3000** **µl** in step 11. 3. Glutathione: Weigh exactly 600 µg glutathione and dissolve in 250 µl sterile Millipore H_2_O, yielding a desired concentration of 2.4 µg/µl. You will use **250** **µl** in step 11. 4. *I*-Inositol: Weigh 225 mg *i*-inositol and dissolve in 5000 µl sterile Millipore H_2_O, yielding a desired concentration of 45 µg/µl. You will use **5000** **µl** in step 11. 5. *O*-Phosphorylethanolamine: Weigh 250 mg *O*-phosphorylethanolamine and dissolve in 3000 µl sterile Millipore H_2_O, yielding a desired concentration of 83.3 µg/µl. You will use **3000** **µl** in step 11. 6. Ribose: Weigh 6.25 mg ribose and dissolve in 1000 µl sterile Millipore H_2_O, yielding a desired concentration of 6.25 µg/µl. You will use **1000** **µl** in step 11. 7. Selenous acid: Weigh 400 µg selenous acid and dissolve in 250 µl sterile Millipore H_2_O, yielding a desired concentration of 1.6 µg/µl. You will use **250** **µl** in step 11. 8. Thiamine hydrochloride: Weigh 4 mg thiamine hydrochloride and dissolve in 1000 µl sterile Millipore H_2_O, yielding a desired concentration of 4.0 µg/µl. You will use **1000** **µl** in step 11. 9. α-Tocopherol phosphate: Weigh 150 µg α-tocopherol phosphate and dissolve in 1000 µl sterile Millipore H_2_O, yielding a desired concentration of 0.15 µg/µl. You will use **1000** **µl** in step 11. 10. Vitamin B12: Weigh 17.5 mg vitamin B12 and dissolve in 2000 µl sterile Millipore H_2_O, yielding a desired concentration of 8.75 µg/µl. You will use **2000** **µl** in step 11. 11. Add the indicated volumes in **bold** to a 50 ml tube. This will result in 17 ml solution. Add 3 ml sterile Millipore H_2_O to obtain a 20 ml solution. 12. Sterile filter. 13. Label sterile Eppendorf tubes with “H_2_O solution”. Mark the lid with an H2. The solution made is for many tubes but we suggest preparing 50 tubes. 14. Aliquot in the sterile Eppendorf tubes (200 µl per tube). 15. Store at −80 °C.

**Table 10:** Preparation of the all-*trans* retinoic acid solution.

Product	Supplemented concentration	Amount in the aliquot (10 µl)
All-*trans*-retinoic acid	25 ng/ml	12.5 µg
**General Information**		
All-*trans*-retinoic acid is highly light sensitive and sensitive to air. Work in subdued light i.e. turn off the light in the LAF bench and dim the light in the room. Do not work with powder or concentrated all-*trans*-retinoic acid if you are pregnant. The compound is teratogenic. Dissolve all retinoic acid. Prepare a stock solution for receiving the supplemented concentration in 500 ml defined medium as shown above.		
**Preparation** 1. Open the glass ampoule with all-*trans*-retinoic acid with care. 2. Dissolve all the powder (50 mg) in 2000 µl of 100 % DMSO (tissue culture grade), yielding a desired concentration of 25 µg/µl. You need to transfer to a sterile test tube. Make sure all powder is dissolved. 3. Add 950 µl of DMSO to a sterile Eppendorf tube. 4. Then add 50 µl of the concentrated solution of all-*trans*-retinoic acid. 5. Label sterile Eppendorf tubes with “Retinoic acid”. Mark the lid with RA. The solution made is for many tubes but we suggest preparing 50 tubes. 6. Aliquot in the sterile Eppendorf tubes (10 µl per tube). 7. Store at −80 °C well protected from light for six months.		
**Note**		
All-*trans*-retinoic acid may not be required for some cell lines that can be induced to differentiate by retinoic acid treatment. However, the all-*trans*-retinoic acid concentration used here is very low (83.3 nM) compared to the concentrations used when inducing differentiation e.g. in SH-SY5Y neuroblastoma cells (1–10 µM).

**Table 11:** Preparation of the β-estradiol solution.

Product	Supplemented concentration	Amount in the aliquot (10 µl)
β-Estradiol	0.5 pg/ml	250 ng
**General Information**		
Prepare a stock solution for receiving the supplemented concentration in 500 ml defined medium as shown above.		
**Preparation** 1. Weigh 100 µg of β-estradiol and dissolve in 400 µl 100 % DMSO, yielding a desired concentration of 0.25 µg/µl. 2. Add 450 µl DMSO to a sterile test tube. 3. Add 50 µl of the DMSO solution with β-estradiol to the tube with 450 µl DMSO. 4. Label sterile Eppendorf tubes with “β-Estradiol”. Mark the lid with bE. 5. Aliquot in the sterile Eppendorf tubes (10 µl per tube). 6. Store the solution at −20 °C. 7. Store the DMSO stock at −80 °C.

**Table 12:** Preparation of the hydrocortisone solution.

Product	Supplemented concentration	Amount in the aliquot (20 µl)
Hydrocortisone	0.25 ng/ml	125 ng
**General Information**		
Prepare a stock solution for receiving the supplemented concentration in 500 ml defined medium as shown above.		
**Preparation** 1. Weigh 1 mg of hydrocortisone in a microcentrifuge tube and dissolve in 2000 µl 99.9 % ethanol, yielding a desired concentration of 0.5 µg/µl. 2. Sterile filter. 3. Add 1975 µl sterile PBS to a sterile Eppendorf tube. 4. Add 25 µl of the hydrocortisone-ethanol solution. 5. Label sterile Eppendorf tubes with “Hydrocortisone”. Mark the lid with an H. 6. Aliquot in the sterile Eppendorf tubes (20 µl per tube). 7. Store at −80 °C.

**Table 13:** Preparation of the triiodothyronine solution.

Product	Supplemented concentration	Amount in the aliquot (10 µl)
Triiodothyronine	0.2 pg/ml	0.1 µg
**General Information**		
Prepare a stock solution for receiving the supplemented concentration in 500 ml defined medium as shown above.		
**Preparation** 1. Weigh 500 µg of triiodothyronine and add 1000 µl 100 % DMSO, yielding a desired concentration of 0.5 µg/µl. 2. Add 490 µl 100 % DMSO to a sterile Eppendorf tube. 3. Add 10 µl of the triiodothyronine solution to the tube with DMSO. 4. Label sterile Eppendorf tubes with Triiodothyronine. Mark the lid with a T. 5. Aliquot in the sterile Eppendorf tubes (10 µl per tube). 6. Store at −80 °C		

## Here you will find the protocols for the protein solution stocks

**Table 14:** Preparation of the basic fibroblast growth factor.

Product	Supplemented concentration	Amount in the aliquot (50 µl)
Basic Fibroblast Growth Factor (bFGF)	1 ng/ml	0.5 µg
**General Information**		
Prepare a stock solution for receiving the supplemented concentration in 500 ml defined medium as shown above.		
**Preparation** 1. Dissolve 25 µg bFGF in 2500 µl sterile Millipore H_2_O, yielding a desired concentration of 0.01 µg/µl. 2. Label sterile Eppendorf tubes with “bFGF”. Mark the lid with a bF. The solution made is for 50 tubes and we recommend aliquoting all the solution. 3. Aliquot in the sterile Eppendorf tubes (50 µl per tube). 4. Store at −80 °C.

**Table 15:** Preparation of the collagen solution.

Product	Supplemented concentration	Amount in the aliquot (100 µl)
Collagen Type 4	100 ng/ml	50 µg
**General Information**		
Prepare a stock solution for receiving the supplemented concentration in 500 ml defined medium as shown above.		
**Preparation** 1. Dissolve 5 mg collagen in 10 ml sterile PBS, yielding a desired concentration of 0.5 mg/ml. 2. Sterile filter. 3. Label sterile Eppendorf tubes with “Collagen”. Mark the lid with a C. The solution made is for 100 tubes and we recommend aliquoting all the solution. 4. Aliquot in the sterile Eppendorf tubes (100 µl per tube). 5. Store at −80 °C.

**Table 16:** Preparation of the epidermal growth factor solution.

Product	Supplemented concentration	Amount in the aliquot (250 µl)
Epidermal Growth Factor (EGF)	10 ng/ml	5 µg
**General Information**		
Prepare a stock solution for receiving the supplemented concentration in 500 ml defined medium as shown above.		
**Preparation** 1. Open the EGF vial carefully. 2. Dissolve 200 µg EGF in 10 ml sterile PBS by adding 0.5 ml PBS at a time to the vial, carefully mixing, and finally transferring the liquid to a 15 ml tube, yielding a desired concentration of 20 µg/ml. 3. Sterile filter the solution. 4. Label sterile Eppendorf tubes with “EGF”. Mark the lid with an E. The volume prepared is for 38–40 tubes depending on loss and we recommend aliquoting all the solution. 5. Aliquot in the sterile Eppendorf tubes (250 µl per tube). 6. Store at −80 °C.

**Table 17:** Preparation of the fetuin A (α2-hs-glycoprotein) solution.

Product	Supplemented concentration	Amount in the aliquot (50 µl)
Fetuin A	40 ng/ml	20 µg
**General Information**		
Prepare a stock solution for receiving the supplemented concentration in 500 ml defined medium as shown above.		
**Preparation** 1. Dissolve 1 mg of fetuin A in 2500 µl sterile PBS, yielding a desired concentration of 0.4 µg/µl. Mix carefully. Do not shake. 2. Sterile filter. 3. Label sterile Eppendorf tubes with “Fetuin A”. Mark the lid with a FA. The volume prepared is for 47–50 tubes depending on loss and we recommend aliquoting all the solution. 4. Aliquot in the sterile Eppendorf tubes (50 µl per tube). 5. Store at −80 °C.

**Table 18:** Preparation of the fibronectin solution.

Product	Supplemented concentration	Amount in the aliquot (500 µl)
Fibronectin	1.33 µg/ml	655 µg
**General Information**		
Fibronectin is tricky to solve. Be very patient. Do never shake! Prepare a stock solution for receiving the supplemented concentration in 500 ml defined medium as shown above.		
**Preparation** 1. Heat sterile Millipore water to 37 °C and warm the bottle with 10 mg fibronectin to 37 °C. 2. Open the bottle carefully and add 7518 µl of 37 °C Millipore water, yielding a desired concentration of 1.33 µg/µl. Put on the lid. 3. Turn the bottle very gently and let the fibronectin slowly dissolve at 37 °C. Do not shake! 4. It may take up to 24 h but just be patient. Turn the bottle gently now and then. 5. Sterile filter. 6. Label sterile Eppendorf tubes with “Fibronectin”. Mark the lid with a F. The solution made is for 15 tubes and we recommend aliquoting all the solution. 7. Aliquot in the sterile Eppendorf tubes (500 µl per tube). 8. Store at −20 °C.		

**Note**		

The thawing of fibronectin must be gentle! Take a tube and keep it in the incubator at 37 °C for 1 h. Do not shake.

**Table 19:** Preparation of PBS with 0.1 % human serum albumin (HSA).

Product
PBS + Human Serum Albumin (HSA)
**General Information**
This is needed to prepare the IGF-1, PDGF, and laminin solutions (see Table 20, Table 21, and Table 22, respectively).
**Preparation** 1. Dissolve 100 mg HSA in 100 ml sterile PBS. Swirl gently to dissolve it. Do not shake. 2. Sterile filter. 3. Store at −20 °C.

**Table 20:** Preparation of the insulin-like growth factor 1 solution.

Product	Supplemented concentration	Amount in the aliquot (25 µl)
Insulin-like growth factor 1 (IGF-1)	5 ng/ml	2.5 µg
**General Information**		
Prepare a stock solution for receiving the supplemented concentration in 500 ml defined medium as shown above.		
**Preparation** 1. Dissolve 100 µg IGF-1 in 1000 µl sterile PBS with 0.1 % HSA in PBS, yielding a desired concentration of 0.1 µg/µl. Mix carefully. Do not shake. 2. Label sterile Eppendorf tubes with “IGF-1″. Mark the lid with an I. The solution made is for 40 tubes and we recommend aliquoting all the solution. 3. Aliquot in the sterile Eppendorf tubes (25 µl per tube). 4. Store at −80 °C.

**Table 21:** Preparation of the platelet-derived growth factor AA solution.

Product	Supplemented concentration	Amount in the aliquot (50 µl)
Platelet-derived growth factor AA (PDGF)	2 ng/ml	1 µg
**General Information**		
Prepare a stock solution for receiving the supplemented concentration in 500 ml defined medium as shown above.		
**Preparation** 1. Dissolve 50 µg of PDGF in 200 µl sterile Millipore water, yielding a desired concentration of 0.25 µg/µl. 2. Add 2300 µl PBS with 0.1 % HSA (see Table 19). 3. Label sterile Eppendorf tubes with PDGF. Mark the lid with a P. 4. Aliquot in the sterile Eppendorf tubes (50 µl per tube). The solution made is for 50 tubes and we recommend aliquoting all the solution. 5. Store at −80 °C.

**Table 22:** Preparation of the laminin solution.

Product	Supplemented concentration	Amount in the aliquot (50 µl)
Laminin	20 ng/ml	10 µg
**General Information**		
Prepare a stock solution for receiving the supplemented concentration in 500 ml defined medium as shown above.		
**Preparation** 1. The desired concentration has to be 0.2 µg/µl. Thus, dilute 1000 µl of the purchased solution (which contains 500 µg of laminin) in 1500 µl sterile PBS with 0.1 % HSA (see Table 19), resulting in a total volume of 2500 µl. 2. Label sterile Eppendorf tubes with “Laminin”. Mark the lid with an L. 3. Aliquot in the sterile Eppendorf tubes (50 µl per tube). The solution made is for 50 tubes and we recommend aliquoting all the solution. 4. Store at −80 °C.

**Table 23:** Preparation of the transferrin solution.

Product	Supplemented concentration	Amount in the aliquot (2.5 ml)
Transferrin	50 µg/ml	25 mg
**General Information**		
Prepare a stock solution for receiving the supplemented concentration in 500 ml defined medium as shown above.		
**Preparation** 1. Open the seal halfway of the bottle containing 1 g of transferrin. 2. Add 10 ml sterile Millipore water to the bottle. Hold the seal down and turn several times. Pipette the solution to a sterile 100 ml bottle. Keep this first pipette in the bottle! Powder may get stuck in this pipette! Take a new 10 ml pipette and add 10 ml sterile Millipore water. Again, hold down the seal and turn several times. Transfer the solution with the 10 ml pipette in the bottle. Repeat until the volume is 100 ml (*i.e*. 10 times 10 ml). 3. Sterile filter. 4. Label sterile 5 ml tubes with ”Transferrin”. 5. Aliquot the transferrin solution (2.5 ml per tube). 6. Store at −20 °C.		

**Caution**		

Be careful not to lose any of the powder. Clumps easily remain stuck inside the pipette used for mixing. To prevent this, use two 10 ml pipettes. One for adding the sterile Millipore H_2_O and one for transferring to the glass bottle. When adding Millipore H_2_O to the bottle with powder, keep the transferring pipette in the 100 ml bottle.

**Table 24:** Preparation of the vitronectin solution.

Product	Supplemented concentration	Amount in the aliquot (200 µl)
Vitronectin	100 ng/ml	50 µg
**General Information**		
Prepare a stock solution for receiving the supplemented concentration in 500 ml defined medium as shown above.		
**Preparation** 1. Label sterile Eppendorf tubes with “Vitronectin”. Mark the lid with a V. 2. Aliquot the vitronectin in sterile Eppendorf tubes (200 µl per tube). 3. Store at −80 °C.

**Table 25:** Preparation of the human serum albumin (HSA) solution for the defined medium.

Product	Supplemented concentration	Amount in the aliquot (20 ml)
Human Serum Albumin (HSA)	1.25 mg/ml	625 mg
**General Information**		
Prepare a stock solution for receiving the supplemented concentration in 500 ml defined medium as shown above.		
**Preparation** 1. Add 300 ml of sterile PBS (at room temperature) in a sterile 500 ml bottle and add 12.5 g of HSA to it. 2. Add another 100 ml of sterile, PBS (at room temperature), yielding a desired concentration of 31.25 mg/ml. 3. Do not shake. Rock the bottle gently until the HSA is dissolved. It may take an hour. Do not shake. 4. Sterile filter. 5. Label sterile 50 ml tubes with “Human Serum Albumin”. 6. Aliquot in the sterile tubes (20 ml per tube). 7. Store at −20 °C.		

**Note**		

Thaw slowly and never shake.		

## Prepare small plastic zip-lock bags with tubes stored at −80 °C

This steps makes it easier to collect the tubes needed when making the defined medium. To avoid thawing, it is recommended to keep all components on dry ice while preparing the bags.

1. Label the bags with:
  - H_2_O
  - NaOH
  - Ethanol
  - Collagen
  - β-Estradiol
  - EGF
  - bFGF
  - Fetuin A
  - Hydrocorisone
  - IGF1
  - Laminin
  - PDGF
  - Retinoic acid
  - Triiodothyronine
  - Vitronectin
2. Add tubes to the bags.
3. Store at −80 °C.

## Ethics statements

There are no ethical considerations regarding this work. No human subjects or animals are used. No data is collected from social media platforms.

## CRediT authorship contribution statement

**Tilo Weber:** Conceptualization, Writing – review & editing. **Jeffrey Bajramovic:** Writing – review & editing. **Stina Oredsson:** Conceptualization, Methodology, Writing – review & editing.

## Data Availability

Data will be made available on request.

## Appendix Supplementary materials

Image, application 1

## Footnotes

1 Milford, Massachusetts, USA. https://www.seracare.com/.

2 Vienna, Austria. https://tht-biomaterials.com/.

3 Corning, New York, USA. https://www.corning.com/.
